# Outer-Membrane-Vesicle–Associated O Antigen, a Crucial Component for Protecting Against *Bordetella parapertussis* Infection

**DOI:** 10.3389/fimmu.2018.02501

**Published:** 2018-10-29

**Authors:** Daniela Bottero, María Eugenia Zurita, María Emilia Gaillard, Francisco Carriquiriborde, Pablo Martin Aispuro, Maia Elizagaray, Erika Bartel, Celina Castuma, Daniela Hozbor

**Affiliations:** ^1^Laboratorio VacSal, Facultad de Ciencias Exactas, Instituto de Biotecnología y Biología Molecular, Universidad Nacional de La Plata, La Plata, Argentina; ^2^Facultad de Ciencias Exactas, Instituto de Estudios Inmunológicos y Fisiopatológicos, Universidad Nacional de La Plata, La Plata, Argentina

**Keywords:** *Bordetella parapertussis*, outer-membrane vesicles, lipopolysacharides, O-antigen, protection

## Abstract

*Bordetella parapertussis* is a respiratory-disease pathogen producing symptomatology similar to that of pertussis but of underestimated incidence and with no specific vaccine existing. We recently designed a vaccine candidate from *B. parapertussis* outer-membrane vesicles (OMVs) that proved to be safe and protective in a murine-infection model. Based on protection recently reported for the *B. parapertussis* O antigen in aqueous solution, we assessed here whether the *B. parapertussis* O-antigen-containing lipopolysaccharide (BppLPS-O^+^) embedded in the membranes, as present in *B. parapertussis*-derived OMVs (OMVs(Bpp-LPS-O^+^)), was the component responsible for that previously observed protection by OMVs. By performing a comparative study with OMVs from a human strain with undetectable O antigen (OMVs(Bpp-LPS-O^−^)), we demonstrated that the OMVs(Bpp-LPS-O^+^), but not the OMVs(Bpp-LPS-O^−^), protected mice against sublethal *B. parapertussis* infections. Indeed, the *B. parapertussis* loads were significantly reduced in the lungs of OMVs(Bpp-LPS-O^+^) -vaccinated animals, with the CFUs recovered being decreased by 4 log units below those detected in the non-immunized animals or in the animals treated with the OMVs(Bpp-LPS-O^−^), (*p* < 0.001). We detected that the OMVs(Bpp-LPS-O^+^) induced IgG antibodies against *B. parapertussis* whole-cell lysates, which immunocomponents recognized, among others, the O antigen and accordingly conferred protection against *B. parapertussis* infection, as observed in *in-vivo*–passive-transfer experiments. Of interest was that the OMVs(Bpp-LPS-O^+^) -generated sera had opsonophagocytic and bactericidal capabilities that were not detected with the OMVs(Bpp-LPS-O^−^)-induced sera, suggesting that those activities were involved in the clearance of *B. parapertussis*. Though stimulation of cultured spleen cells from immunized mice with formulations containing the O antigen resulted in gamma interferon (IFN-γ) and interleukin-17 production, spleen cells from OMVs(Bpp-LPS-O^+^) -immunized mice did not significantly contribute to the observed protection against *B. parapertussis* infection. The protective capability of the *B. parapertussis* O antigen was also detected in formulations containing both the OMVs derived from *B. pertussis* and purified BppLPS-O^+^. This combined formulation protected mice against *B. pertussis* along with *B. parapertussis*.

## Introduction

*Bordetella parapertussis* is a species quite close to *Bordetella pertussis* that can infect humans causing similar symptoms to those of the respiratory disease referred to as pertussis. The detection of this pathogen in pertussis patients is relatively frequent in different countries of Europe (1, 2) and also in the USA (3–5). In the latter country, the highest number of *B. parapertussis* infections was recorded in Wisconsin (at 443 cases) between October 2011 and December 2012 (5). As had occurred previously, during such outbreaks 11.2% of the diagnostic specimens positive for *Bordetella* were also positive for *B. parapertussis*, probably indicating the endemnism of *B. parapertussis*. In fact, in the USA *B. parapertussis* was estimated to have caused 16% of the cases diagnosed as pertussis (6). In several countries of Latin America, infections caused by *B. parapertussis* have been detected, but unfortunately no official reports about the incidence rates are available. We wish to note here that in general the global incidence of *B. parapertussis* is probably underestimated, not only in Latin-American countries but also in most others because the official notification of the infections caused by this pathogen are not mandatory. Furthermore, many laboratories do not have the technologic wherewithal to discriminate between *B. pertussis* and *B. parapertussis* infections. In addition, *B. parapertussis* must clearly be recognized as the cause of a pertussis-like disease for which no specific effective preventive strategies have as yet been developed. Moreover, the currently used vaccines for pertussis are not adequate for reducing *B. parapertussis* infections (7). Several of the protective immunogens included in the pertussis vaccines, though homologous to *B. parapertussis* proteins, are antigenically distinct (7, 8). This may be one of the reasons that could explain the observed incomplete cross-protection of pertussis vaccines against *B. parapertussis*. Furthermore, these two species show differences in the expression of components that are essential for the protection induced by pertussis vaccines. As an example, the pertussis toxin is only expressed by *B. pertussis* and since this toxin is not present in *B. parapertussis*, the protective immune response triggered by the toxin has no target in *B. parapertussis*.

Only two studies have indicated that pertussis acellular vaccines could have any effect whatsoever on the control of *B. parapertussis*; and as described by the authors, those studies had several limitations—mainly as a consequence of the underreporting of *B. parapertussis*, which underestimation must necesarily affects the assessment of vaccine effectiveness (2, 9). Even worse, Long et al., using a rodent model of bordetellosis, demonstrated that pertussis acellular vaccination enhances the performance of *B. parapertussis* (10).

The ongoing research activity on *B. parapertussis* points to the demand for a specific vaccine against this pathogen. The proteins of *B. parapertussis*—such as the filamentous hemagglutinin and the iron-transport protein AfuA, among others—had been proven to confer protection against *B. parapertussis* infection in a mouse model (11, 12). In previous work, we started to investigate the potential of outer-membrane vesicles (OMVs) derived from *B. parapertussis* as an alternative approach to a vaccine candidate against *B. parapertussis* (13). Using the mouse model of intranasal infection, we observed that the formulations based on these OMVs efficiently protected mice against *B. parapertussis* infection, whereas current commercial acellular pertussis vaccines exhibited little protection against that particular pathogen (13). OMVs are naturally released by various Gram-negative bacteria and contain predominantly outer-membrane components, including the lipopolysaccharide (LPS), along with periplasmic compounds (14). That the isolated LPS of *B. parapertussis*—and in particular the LPS containing the O antigen (BppLPS-O^+^)—has been found to protect against *B. parapertussis* infection is noteworthy (15). That protection was revealed by the assays carried out in a mouse model in which immunizations were performed with commercial acellular vaccines supplemented with an aqueous solution containing 10 μg of purified BppLPS-O^+^ (15). That protective capability was, in fact, associated specifically with the presence of the O antigen (15). At this point, we must stress that though all *B. parapertussis* has the unusual lipid A structure characteristic to *Bordetella* [absence of symmetry at the C-3 and C-3**'** positions, phospate groups modified with glucosamine and hipo-acylation, (16, 17)], not all lineages of *B. parapertussis* contain an LPS whose structure includes the O antigen. The lineage that infects only humans contains an LPS with the O antigen, whereas the LPS of the strains that have been recovered from sheep lack that antigen (18). We were also interested to note that the isolates containing LPS without the O antigen are highly sensitive to murine complement-mediated killing *in vitro*, and because of that vulnerability those *B. parapertussis* isolates failed to colonize naïve mice (18).

Within this context, in the present work, we assessed whether the *B. parapertussis* LPS with the O antigen embedded in the membranes—as occurs in the example of the OMVs derived from *B. parapertussis* OMVs(Bpp-LPS-O^+^)—would prove to be the crucial component for the previously reported protection of the OMVs (13). To assess the role of LPS-O^+^, the immunity and protection conferred by *B. parapertussis*-OMV vaccination were compared to the properties generated by the OMVs derived from an ATCC strain of *B. parapertussis* in which the O antigen was not detected (OMVs(Bpp-LPS-O^−^)). Mice immunized with OMVs derived from a clinical isolate of *B. parapertussis* whose LPS contained the O antigen were protected against *B. parapertussis*, but not those treated with OMVs derived from *B. parapertussis* lacking that antigen. By performing *in-vitro* and *in-vivo* experiments, we detected that the humoral response containing O-antigen-specific antibodies contributed to the protection induced by the OMVs(Bpp-LPS-O^+^) -vaccines. In contrast, sera collected from OMVs(Bpp-LPS-O^−^) immunized mice produced no such reduction of *B. parapertussis* colonization upon passive transfer. Purified BppLPS-O^+^, but not the BppLPS-O^−^, induced protection in mice against *B. parapertussis* challenge. Moreover, the inclusion of BppLPS-O^+^, but not BppLPS-O^−^, rendered the vaccine based on OMVs derived from *B. pertussis* (19–24) efficacious against both *B. parapertussis* and *B. pertussis* challenge. All together, these data indicated that LPS-O^+^ is a crucial protective antigenic component of the OMVs derived from *B. parapertussis*.

## Materials and methods

### Bacterial strains and growth conditions

*Bordetella parapertussis* AR729, an Argentine clinical isolate previously used to obtain the OMVs, along with the ATCC strain *B. parapertussis* 15237—the isolate from Eldering and Kendrick lacking the LPS O antigen—were used throughout this study (25). In contrast to other human *B. parapertussis* isolates, the ATCC strain has undetectable O antigen (18). These strains were grown either in Bordet-Gengou agar (BGA, Difco) supplemented with 1% (v/v) glycerol, 10 g/L Bacto peptone (Difco), and 10% (v/v) defibrinated sheep blood and incubated at 36°C, or alternatively in Stainer-Scholte liquid medium (26), as indicated previously (13).

### Isolation of OMVs

To obtain OMVs from the bacterial cells, we used the method previously described by us (19, 20, 27). The procedure stated in brief: Culture samples from the decelerating growth phase of the bacteria were centrifuged at 10,000 x g for 20 min at 4°C and the pellet obtained resuspended in 20 mM Tris-HCl, 2 mM EDTA, pH 8.5 (TE Buffer). Of the resulting pellet, ~1 g (wet weight) was resuspended in 5 mL of the TE Buffer. OMV release was promoted by sonication in ice-water; the cells were then removed by centrifugation at 10,000 x g and the OMV-containing supernatant concentrated by ultracentrifugation at 40,000 x g for 3 h. The OMVs thus obtained were stored at 4°C. Thereafter the OMVs were examined by electron microscopy after negative staining (20).

### Protein assay

The protein content was estimated by the Bradford method with BSA as a standard (28).

### LPS sodium dodecylsulfate-polyacrylamide gel electrophoresis (SDS-PAGE) and immunoblotting

The LPS from the OMVs of *B. parapertussis* was solubilized in Laemmli sample buffer (29) and heated at 100°C for 10 min. Twenty-five mg of proteinase K in 10 μL of Laemmli sample buffer were added per 50 μL of OMV suspension. The mixtures were incubated in a water bath at 60°C for 1 h with occasional vortexing. The proteinase-K–treated samples were applied to the gels and the electrophoresis performed at room temperature and constant voltage. The LPS visualized by the BioRad silver-staining technique was transferred to polyvinylidene-difluoride membranes (Immobilon P; Millipore) and probed with OMV-immune sera (1:2,000) followed by incubation with anti(mouse IgG) conjugated with alkaline phosphatase at a 1:1,000 dilution. Nitroblue tetrazolium and 5-bromo-4-chloro-3-indolyl-phosphate were used as the phosphatase substrates according to the manufacturer's protocol (Biodynamics SRL Buenos Aires Argentina).

### Isolation of *B. parapertussis* LPS

The LPS from *B. parapertussis* was isolated by the hot phenol-water method, along with previously described modifications (30). The samples of LPS were dialyzed and lyophilized. Dry weights were used to determine the amounts of LPS obtained. The quality of each sample was checked by SDS-polyacrylamide gel electrophoresis (SDS-PAGE).

### Formulation of vaccines

To use the OMVs or purified LPS as acellular vaccines, vesicle (3 μg) and LPS (1 μg) preparations were detoxified by mixing with aqueous formaldehyde (0.37% [v/v]) and incubating at 37°C overnight, with aluminum hydroxide (0.2 mg/mL) thereafter being added as an adjuvant.

### Active immunization and intranasal challenge

Four-week-old female BALB/c mice (*n* = 12 per experimental group) obtained from Biol SAIC, Argentina were used for all assays. As described previously (19), the immunization protocols comprised a two-dose schedule (i.p.) with the formulations described above over a period of 2 weeks. Two weeks after the second immunization, the mice were subjected to a nasal challenge with a sublethal dose (10^6^-10^7^ CFUs in 40 μL) of *B. parapertussis* strain AR729. Then, at 7 days after the challenge, the lungs of the mice were excised and collected for bacterial counts. The number of CFUs was determined as previously described (19). These experiments were carried out in accordance with the recommendations of the Guide for the Care and Use of laboratory animals, National Research Council of National Academies, Washington DC 2010 and/or Directive 2010/63/EU on the protection of animals. The protocol was approved by the Ethical Committee for Animal Experiments of the Faculty of Science at La Plata National University (approval number 003-06-15).

### Enzyme-linked immunosorbent assay (ELISA)

Plates were coated with sonicated *B. parapertussis* whole-cell lysates in 0.5 M carbonate buffer, pH 9.5 in an overnight incubation at 4°C and then blocked with 3% (v/v) skimmed milk in blocking buffer (2 h at 37°C) before incubation with serially diluted mouse-serum samples (1 h at 37°C). The bound IgG was detected after a 2-h incubation with horseradish-peroxidase–conjugated goat anti(mouse IgG) at a titer of 1:20,000 (Thermo Fisher Scientific, Buenos Aires Argentina). For measuring IgG isotypes, the detection of bound antibody was determined with horseradish-peroxidase–labeled subclass-specific anti(mouse IgG1 or IgG2a) at 1:8,000 or 1:1,000, respectively (Sigma Aldrich, USA). The substrate used was 1.0 mg/mL o-phenylendiamine (Bio Basic Canada Inc.) in 0.1 M citrate-phosphate buffer, pH 5.0 containing 0.1% (v/v) hydrogen peroxide. The optical densities (ODs) were measured with a Titertek Multiskan Model 340 microplate reader (ICN, USA) at 492 nm and the OD plotted as a function of the log of the reciprocal serum dilution. The inflection point of the curve was determined by the GraphPadPrims® software. Titers were defined as the reciprocal serum dilution giving an OD corresponding to the inflection point of the curve.

### Cell-line growth

Macrophage-like RAW 264.7 cells (ATCC TIB-71) were grown in Roswell Park Memorial Institute (RPMI) 1640 medium containing 10% (v/v) heat-inactivated fetal-bovine serum (FBS) and an antibiotic solution of 100 international units (I U)/mL penicillin and 100 μg/mL streptomycin at 37°C in 5% (v/v) CO_2_/air.

### Opsonophagocytosis assay

A *B. parapertussis* strain expressing the green-fluorescent protein (GFP)—the *B. parapertussis* AR729 carrying plasmid pCW504, kindly provided by Dr. A. Weiss—was opsonized by incubation at 37°C with different serum samples added at 20% (v/v) for 30 min in a final volume of 40 μL. Serum-opsonized GFP-expressing bacteria were incubated with RAW 264.7 cells at a multiplicity of infection of 70 for 30 min at 37°C to enable binding and internalization. After extensive washing to remove non-attached bacteria, the samples were analyzed with a FACS Calibur flow cytometer (Becton Dickinson, San Jose, CA) and the results of the estimated degree of phagocytosis expressed as the mean fluorescence intensity. Statistical significance was assessed by the one-way analysis of variance (ANOVA) followed by Bonferroni's multiple-comparison test (GraphPadPrims®). Differences were considered to be significant when *p* < 0.001.

### Bactericidal assay

The bactericidal activity of the sera collected from mice 2 weeks after immunization with the OMV(Bpp-LPS-O^+^) and OMV(Bpp-LPS-O^−^) vaccines (i.e., the immune sera) and from non-immunized mice (i.e., the naïve sera) were tested *in vitro*. Naïve sera from mice were used as a source of complement. Both heat-inactivated immune and naïve sera and the vehicle phosphate-buffered saline (PBS) alone were used as controls. Virulent *B. parapertussis* were grown on Bordet Gengou agar medium and diluted to 1 × 10^5^ CFUs/mL in PBS containing MgCl_2_ 0.05 M and CaCl_2_ 0.15 mM. Forty-five microliters of heat inactivated serum or PBS were mixed with 5 μL of a suspension containing 500 CFUs of the bacteria. After 1 h of incubation at 37°C, 5 μL of naïve serum was added to each of the samples tested. After 1 h of incubation at 37°C, serial dilutions of the samples were spread on Bordet Gengou agar plates and incubated for 48–72 h to determine the CFUs. At least three biological replicates were performed.

### Analysis of the cellular response elicited by vaccination

The cellular response was analyzed as previously described (21, 31). Briefly, spleen cells from mice immunized with the OMV-based vaccines were harvested 8 weeks after the last immunization and seeded in 48-well culture plates at 10^6^ per well in a volume of 500 μL of RPMI 1640 cell-culture medium supplemented with 10% (v/v) fetal-bovine serum (Invitrogen, Buenos Aires Argentina) containing 100 IU/mL penicillin and 100 μg/mL streptomycin. All the spleen cells were either stimulated with OMVs derived from *B. parapertussis* (5 μg/mL) or exposed to medium alone. Supernatants were removed after 72 h of incubation at 37°C in an atmosphere of 5% (v/v) CO_2_/air and the production of interferon-γ (IFN-γ), and IL-17 determined by ELISA (BD Biosciences, CA USA), according to the conditions specified by the manufacturer.

### Adoptive transfer

Pooled sera (100 μL) or spleen cells (5 × 10^7^) from non-immunized mice or from mice immunized with the OMV- or LPS-based vaccine 2 weeks previously were transferred intraperitoneally to female BALB/c mice (*n* = 12). Twenty-four h thereafter, the mice were infected with a sublethal dose (10^6^-10^7^ CFUs 40 in μL) of *B. parapertussis* AR729 and the subsequent protection assessed by determining the CFU counts in the mouse lungs 7 days after the challenge.

### Statistical analysis

The data were evaluated statistically by the Student *t*-test or the ANOVA followed by the Bonferroni *post-hoc* test (via the GraphPad Prism® software) as indicated in the legends to the figures. Differences were considered significant at a *p* < 0.05.

## Results

### Isolation and characterization of the OMVs obtained from *B. parapertussis* AR729 and ATCC 15237 strains

**Figure 1:**
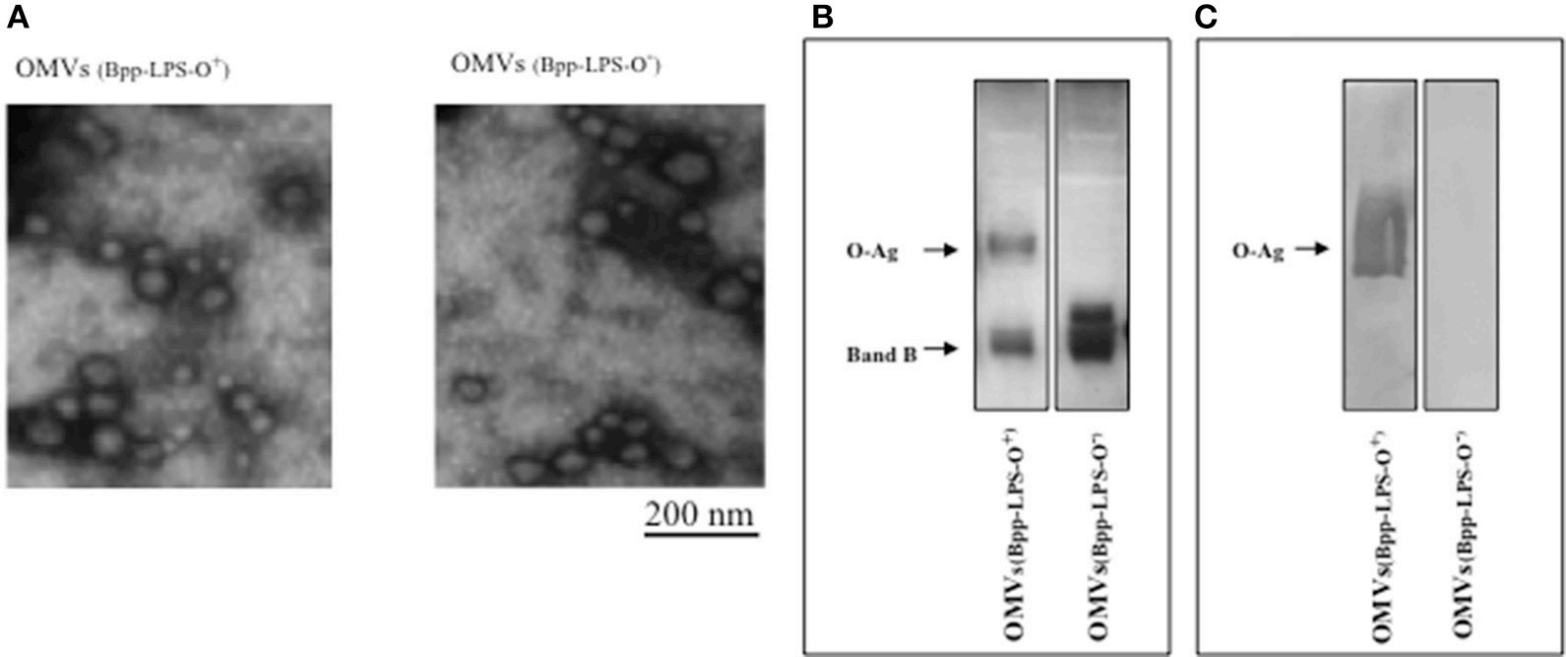
Characterization of OMVs by electron microscopy and electrophoresis**. (A)** Transmission-electron-microscopy image of the negatively stained preparations obtained in the virulent phase from *B. parapertussis* strains AR729 (OMVs(Bpp-LPS-O^+^)) and ATCC 15237 (OMVs(Bpp-LPS-O^−^)). Scale bar: 200 nm. **(B)** LPS analysis of OMVs(Bpp-LPS-O^+^) and OMVs(Bpp-LPS-O^−^) by 15% (w/v) SDS-PAGE. The bands were visualized by the BioRad silver-staining technique. In the figure, the sources of the samples are indicated either above **(A)** or below **(B)** the representative illustrations. **(C)** Immunoblotting of purified *B. parapertussis* LPS separated by 15% (w/v) SDS-PAGE probed with the specific O antigen polyclonal antiserum obtained from mice. The arrows indicate the locations of the O antigen (O-Ag) and the Band B.

The OMV samples obtained from *B. parapertussis* AR729 and *B. parapertussis* ATCC 15237 grown in the virulent phase were negatively stained and examined by electron microscopy (Figure 1A). The procedure was repeated at least four times, and in all the samples the size range (at dimensions of 50–150 nm) was both consistent from batch to batch and similar to that previously described for OMV preparations derived from *B. pertussis* (19) and *B. parapertussis* (13). To characterize the LPS in both OMVs, one-dimensional electrophoresis was performed (Figure 1B). By this technique, we detected that the LPS present in the OMVs preparation derived from *B. parapertussis* AR729 had the characteristic electrophoretic profile of the LPS belonged from human strains of *B. parapertussis* (16, 32) consisting in a faster-migrating LPS—B band that corresponds to the Lipid A- core structure together with a ladder arrangement of high molecular mass bands that correspond to the O antigen (O-Ag, a polymer of 2,3-diacetamido-2,3-dideoxy-α-galacturonic acid (33)). The presence of the O-Ag band in the LPS from AR729 was confirmed by an immunoblot assay using a polyclonal serum that only recognized the O antigen (Figure 1C). In contrast, the LPS from the OMVs derived from the ATCC 15237 strain did not contain the characteristic O-Ag band. Moreover, the polyclonal serum against O antigen did not react with this LPS derived from ATCC15237 strain (Figure 1C).

### Protection against intranasal *B. parapertussis* challenge after vaccination with OMVs obtained from different strains

**Figure 2:**
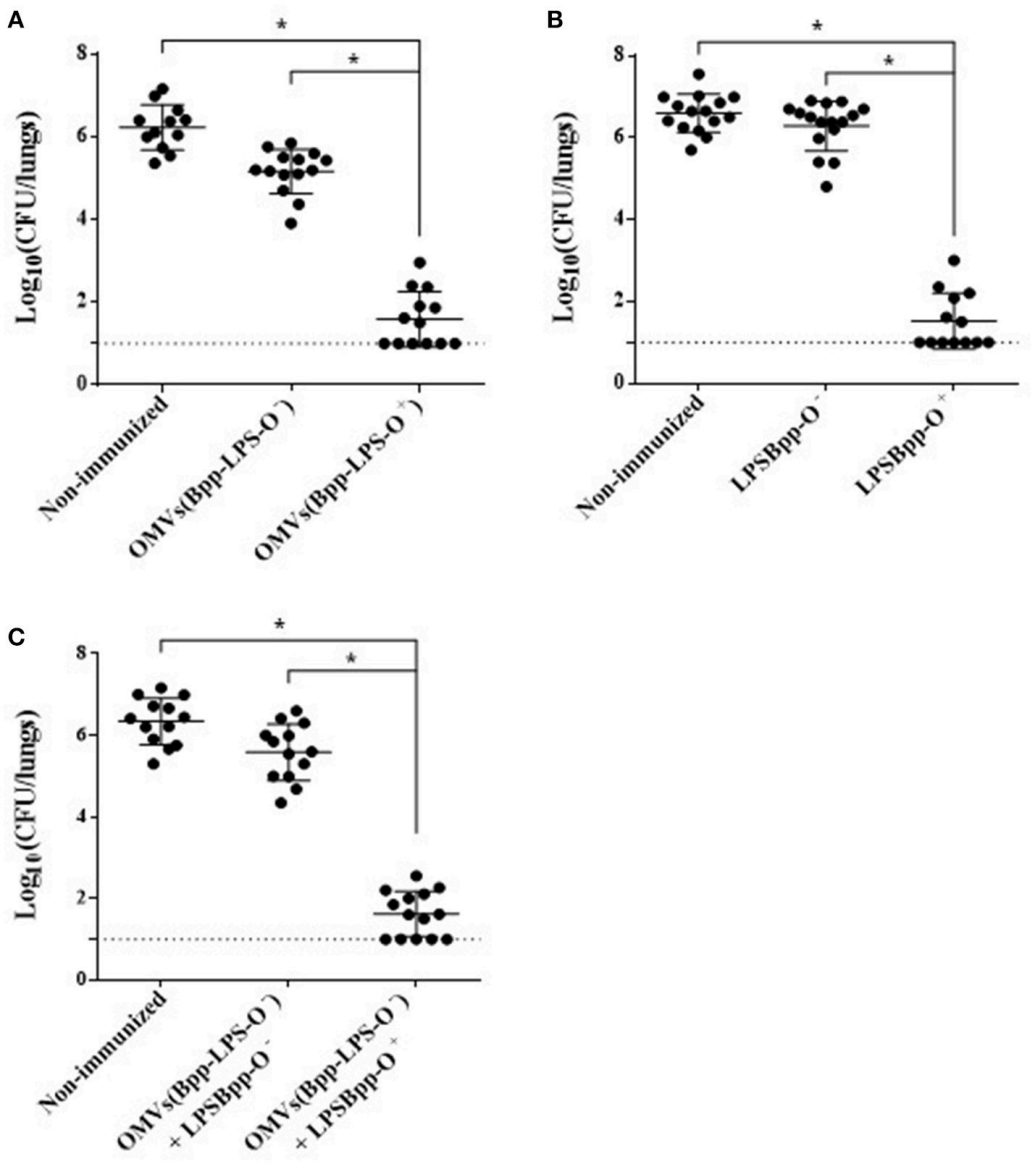
Effect of active immunization on the protection of mice against *B. parapertussis* infection. **(A)** Groups of animals were immunized with OMVs(Bpp-LPS-O^+^), OMVs(Bpp-LPS-O^−^) or non-immunized. **(B)** Groups of animals were immunized with BppLPS-O^+^, BppLPS-O^−^ or were non-immunized. **(C)** Groups of animals were immunized with OMVs(Bpp-LPS-O^−^) supplemented with BppLPS-O^+^, OMVs(Bpp-LPS-O^−^) supplemented BppLPS-O^−^ or were non-immunized. For all panels, the challenge was performed with *B. parapertussis* AR729 (5 x 10^7^ CFUs in 40 μL) as the pathogen. In each of the three panels, the number of bacteria recovered from the mouse lungs, expressed as the log_10_ of the means ± SEM (error bars) of the CFUs per lungs, is plotted on the *ordinate* for the samples from the lungs of the experimental groups indicated on the *abscissa*. Graphs show values for individual mice and the median value (bar, *n* = 12 mice) at 7 days after challenge. The dotted horizontal line marks the lower limit of detection. The asterisks (*) indicate significant differences at a *p* < 0.001 (ANOVA followed by the Bonferroni *post-hoc* test).

To evaluate the protective capability against *B. parapertussis* challenge of the OMVs obtained either from *B. parapertussis* AR729 (OMVs(Bpp-LPS-O^+^)) or *B. parapertussis* ATCC 15237 (OMVs(Bpp-LPS-O^−^)), we used the murine model of intranasal infection (*n* = 12 mice per experimental group). The CFUs recovered from the lungs of the immunized mice were compared with those detected in non-immunized mice (Figure 2). Significant differences were obtained in the lung bacterial counts between the OMVs(Bpp-LPS-O^+^)-immunized and non-immunized mice (*p* < 0.001; Figure 2A) and also between the OMVs(Bpp-LPS-O^+^) -immunized and the OMVs(Bpp-LPS-O^−^) -immunized mice. The number of colonies recovered from the lungs of the OMVs(Bpp-LPS-O^+^) -immunized mice at Day 7 after challenge was at least 4.5 logs lower than those detected in the other two groups of animals included in the assays (Figure 2A).

We next sought to investigate if the different protective capabilities detected for the two OMVs studied here correlated with the corresponding LPSs purified from those OMVs from the two strains of *B. parapertussis*. To that end, we performed *in-vivo* protection assays using the LPSs isolated from the OMVs derived from each strain, BppLPS-O^+^ and BppLPS-O^−^, as vaccines. Mice (*n* = 12 per each group) were accordingly immunized twice with the purified LPSs (1 μg) and then challenged 2 weeks after the second immunization with a sublethal dose of *B. parapertussis* AR729 strain, with non-immunized mice being used as a negative control. For the LPS-O^+^ used in the immunization schedule, but not for the LPS-O^−^, highly significant differences of more than 4.5 logs in the lung *B. parapertussis* colony-formation counts were obtained between the LPS-O^+^ immunized animals and the negative control group or the LPS-O^−^-immunized animals (*p* < 0.001; Figure 2B). Consistent with the role of the O antigen in conferring immune protection, when the purified LPS-O^+^ was added to the formulation based on the OMVs(Bpp-LPS-O^−^), protection against *B. parapertussis* infection was restored to the levels detected for the OMVs(Bpp-LPS-O^+^) vaccine (Figure 2C). As would be expected, no such restoration occurred when the same trial was performed with the purified LPS-O^−^ (Figure 2C).

Since the *B. parapertussis* ATCC 15237 failed to colonize the lungsof mice, protection assays using this strain for the challenge were not performed. The failure in colonization of this strain containing an LPS without the O antigen was expected based on the results reported by Zhang et al. (15).

### Key role of the humoral immune response induced by the OMVs(Bpp-LPS-O+) that recognized, among others, the O antigen in the protection against *B. parapertussis*

**Figure 3:**
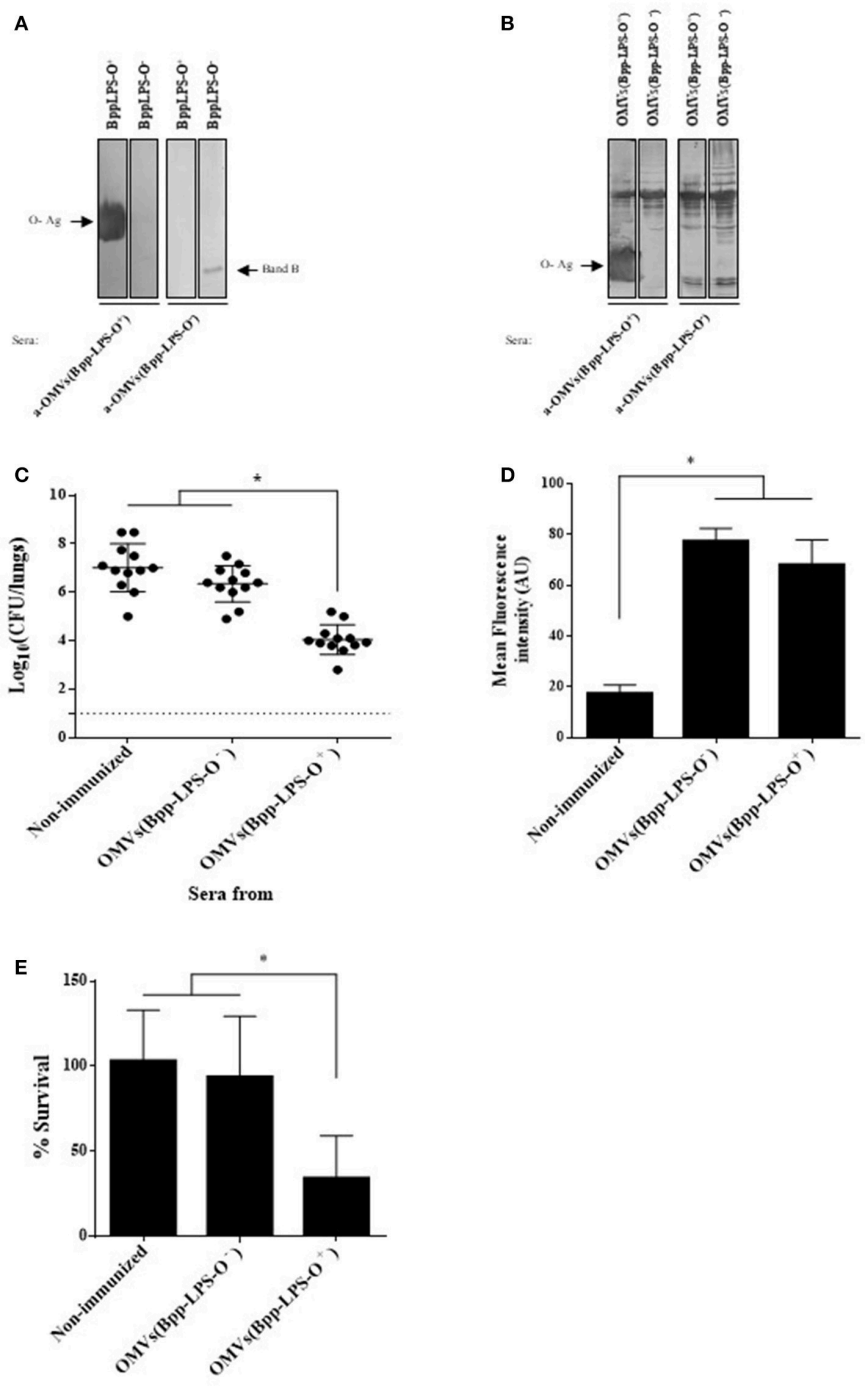
Humoral response induced by purified BppLPS-O^+^ and OMVs(Bpp-LPS-O^+^). **(A)** Immunoblotting of purified *B. parapertussis* LPS separated by 15% (w/v) SDS-PAGE probed with the polyclonal antiserum obtained from mice immunized with OMVs(Bpp-LPS-O^+^) and OMVs(Bpp-LPS-O^−^*)*. The arrows indicate the locations of the O antigen (O-Ag) and the Band B that were recognized by the antiserum used in the subsequent immunoblotting indicated below the gels. These sera are designated according to the immunogen— OMVs(Bpp-LPS-O^+^) and OMVs(Bpp-LPS-O^−^)—used to raise the immune response in the donor mice. **(B)** Immunoblotting of OMVs(Bpp-LPS-O^+^) and OMVs(Bpp-LPS-O^−^) samples separated by 12.5% (w/v) SDS-PAGE and probed with the polyclonal antiserum obtained from mice immunized with OMVs(Bpp-LPS-O^+^) and OMVs(Bpp-LPS-O^−^). The arrows indicate the locations of the O antigen (O-Ag) and the Band B that were recognized by the antiserum used in the immunoblotting indicated below the gels. These sera are designated according to the immunogen—OMVs(Bpp-LPS-O^+^) and OMVs(Bpp-LPS-O^−^)—used to raise the immune response in the donor mice. **(C)** Effect of passive immunization with sera collected from OMVs(Bpp-LPS-O^+^) - or OMVs(Bpp-LPS-O^−^) -immunized mice. Pooled sera from either OMVs(Bpp-LPS-O^+^) - or OMVs(Bpp-LPS-O^−^) -immunized mice were tested by transfer to naive female mice (*n* = 12). Pooled sera from non-immunized mice transferred to naive female mice were used as a negative control of protection. Twenty-four h after the transfer, the mice were infected with *B. parapertussis* AR729 as the challenge bacterium (5 × 10^7^ CFUs in 40 μL). In the figure, the number of bacteria recovered from the mouse lungs, expressed as the log_10_ of the means ± SEM (error bars) of the CFUs per lungs, is plotted on the *ordinate* for the non-immune and the two immune sera indicated on the *abscissa*, with the latter being designated according to the immunogen—OMVs(Bpp-LPS-O^−^) and OMVs(Bpp-LPS-O^+^) —used to raise the immune response in the donor mice. The asterisk (*) indicates a significant difference vs. the naïve sera (*p* ≤ 0.05). **(D)** Opsonophagocytic activiy of the immune sera. The mean fluorescence intensity in arbitrary units (AU) associated with macrophages previously incubated with GFP-expressing *B. parapertussis* AR729 is plotted on the *ordinate* for each of the sera used for opsonization of those bacteria on the *abscissa*, indicated as non-immune or immune, with the latter being designated according to the immunogen—OMVs(Bpp-LPS-O^−^) and OMVs(Bpp-LPS-O^+^)—used to raise the immune response in the donor mice. The asterisk (*) denotes *p* ≤ 0.0001 vs. the intensity obtained after exposure of the bacteria to the non-immunized serm. **(E)** Serum-complement–mediated killing. Sera from OMVs(Bpp-LPS-O^+^)—or OMVs(Bpp-LPS-O^−^)-immunized mice were incubated with *B. parapertussis* AR729 (500 CFU), and 1 h later the bacteria were plated to determine viability. In the figure, the percent survival is plotted on the *ordinate* for the non-immune and the two immune sera indicated on the *abscissa*, with the latter being designated according to the immunogen—OMVs(Bpp-LPS-O^−^) and OMVs(Bpp-LPS-O^+^)—used to raise the immune response in the donor mice. The asterisk (*) indicates a significant difference vs. the naïve sera (*p* ≤ 0.05).

To characterize the humoral immune response induced by the OMV based vaccine that induced protection against *B. parapertussis* [OMVs(Bpp-LPS-O^+^)], we first determined the total IgG and IgG isotypes titers by ELISA assays. The ELISA results showed that mice immunized with OMVs(Bpp-LPS-O^+^) produced high titers of IgG (1,460 ± 210) with an IgG1/IgG2a ratio >1 (the species IgG1 = 656 ± 136 and IgG2a = 315 ± 83; therefore IgG1/IgG2a = 2.08). Then and based on previously reported role of the O-Ag antibodies in protection (15) we wanted to ascertain whether the LPS-O^+^ embedded in the OMVs(Bpp-LPS-O^+^) was able to induce specific antibodies against the O-Ag and, as a consequence, the induced immune sera have protective capability against *B. parapertussis* infection. To this end, we performed immunoblotting assays using purified *B. parapertussis* LPS-O^+^ from *B. parapertussis* AR729 strain as the antigen and LPS-O^−^ from *B. parapertussis* ATCC 15237 for comparison, and probed against sera from OMVs(Bpp-LPS-O^+^) and OMVs(Bpp-LPS-O^−^) immunized mice. Significantly, in this assay the OMVs(Bpp-LPS-O^+^) immune sera recognized a diffuse band that corresponded specifically to the LPS containing the O antigen (Figure 3A, left lane). This band was not detected in the O-antigen–deficient LPS isolated from the *B. parapertussis* ATCC 15237 strain (Figure 3A). In contrast, the sera from OMVs(Bpp-LPS-O^−^)-treated mice recognized only the fast-migrating Band B (Figure 3A, right lane). Naïve sera did not react with either of the bands detected in the SDS-PAGE for both LPSs (not shown). When the OMV-generated immune sera were used in immunoblotting assays, in which the OMVs from both the O-antigen-positive and -negative *B. parapertussis* strains were used as antigens, the polypeptide profile recognized was similar for both sera. The major difference observed between the two sera and the two OMV preparations, was the diffuse band corresponding to the O antigen detected in the OMVs(Bpp-LPS-O^+^) sample by the OMVs(Bpp-LPS-O^+^) sera (Figure 3B, leftmost lane).

With the immune sera induced by OMVs(Bpp-LPS-O^+^) having been characterized in this manner, serum-transfer experiments were performed. The sera to be tested were adoptively injected into naïve animals (*n* = 12) 24 h before an intranasal challenge with *B. parapertussis* (5 × 10^7^ CFUs *B. parapertussis* AR729). Sera from OMVs(Bpp-LPS-O^−^) immunized mice and from naïve mice were used as negative control of protection. Through this assay, we detected that *B. parapertussis* was cleared from the mouse lungs by Day 7 after the inoculation with the OMVs(Bpp-LPS-O^+^) sera containing anti-O Ag antibodies (at a reduction of 4 logs), whereas the OMVs(Bpp-LPS-O^−^) or naïve sera in which anti-O Ag antibodies were not detected had no significant effect (Figure 3C).

To elucidate the mechanisms involved in the protective capacity of the OMVs(Bpp-LPS-O^+^) immune sera, we decided to perform an oposonophagocytosis assay since this activity was previously associated with the presence of antibodies against the *B. parapertussis* O antigen (15). Consistent with the ELISA results demonstrating that mice immunized with OMVs(Bpp-LPS-O^+^) produced high titers of IgG with an IgG1/IgG2a ratio >1, *B. parapertussis* was efficiently opsonized by the antibodies from OMVs(Bpp-LPS-O^+^) -induced serum (Figure 3D). We need to remark however, that the immune sera from OMVs(Bpp-LPS-O^−^) -treated mice also presented oposonophagocytic activity. These results are not surprising since other opsonins are expected to be induced by OMVs because of the wide variety of immunogens present. No significant internalization was detected for bacteria opsonized with naïve serum (Figure 3D).

To determine whether the different degree of protection observed for the two OMV vaccines was correlated with a differential bactericidal capability between the sera induced by both OMVs, killing assays were performed. For these determinations, a suspension of 500 CFUs of *B. parapertussis* bacteria was incubated in 50 μL of 90% (v/v) serum in PBS to insure that the serum components were not limiting. These assays revealed that the *B. parapertussis* bacteria were sensitive to the immune sera induced by the OMVs(Bpp-LPS-O^+^), with only 24.6 ± 10.2% surviving, but resistant to OMVs(Bpp-LPS-O^−^) and naïve sera, with respective percent survivals of 92.8 ± 20.5 and 92.8 ± 11.2 (Figure 3E). In control experiments using heat-inactivated sera, we observed a 100% bacterial survival for both the immune and the naïve sera.

All the results presented here indicated that the presence of O-Ag embedded in the OMVs(Bpp-LPS-O^+^) induced humoral immune responses that conferred a definitive protection against *B. parapertussis*. The protective capability observed seems to be mainly a consequence of the bactericidal activity of the immune sera.

**Figure 4:**
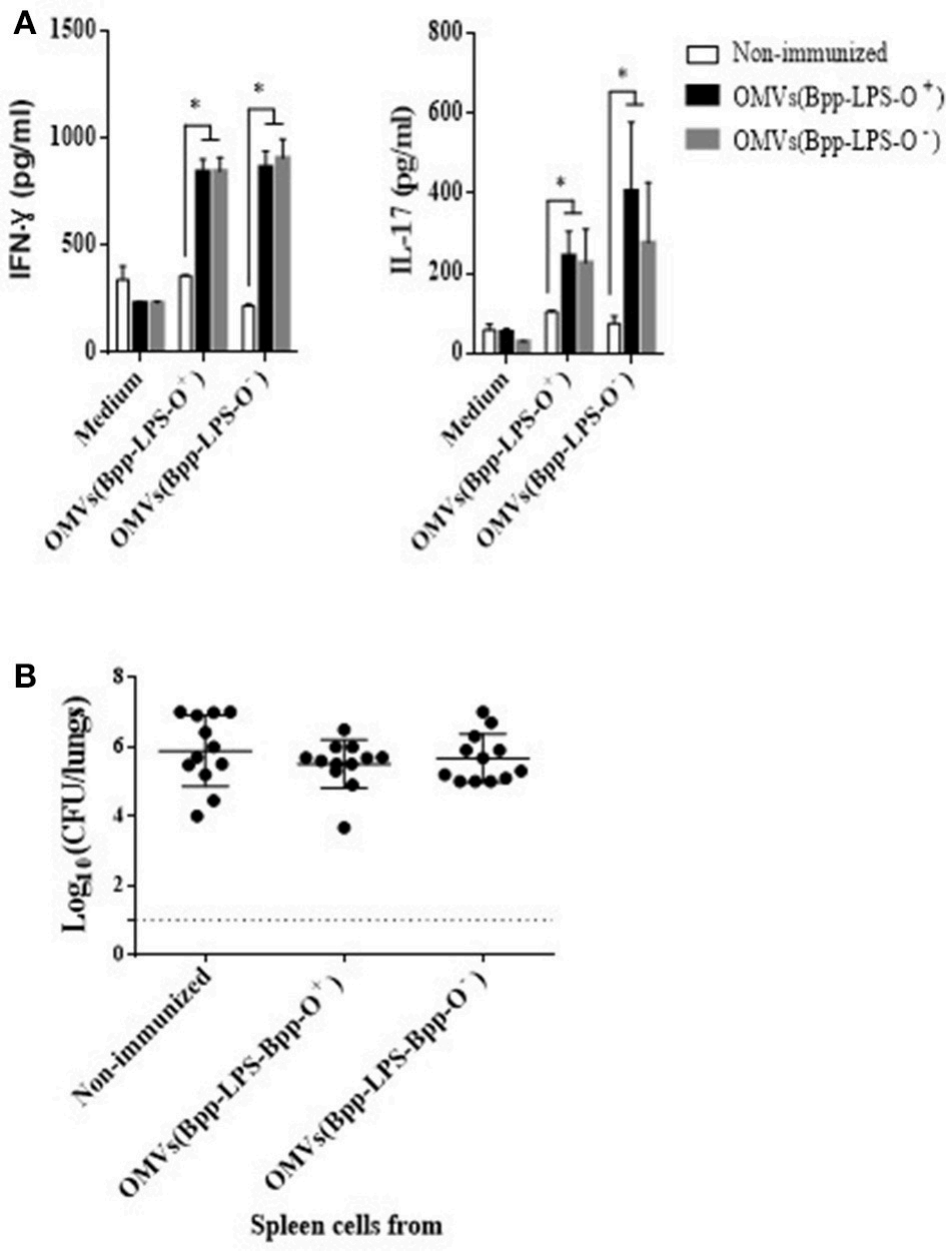
Immune response induced by active immunization with OMVs(Bpp-LPS-O^+^) vaccine. **(A)** Cytokine production by spleen cells from immunized mice. BALB/c mice were non-immunized or immunized with two doses of OMVs(Bpp-LPS-O^+^) or OMVs(Bpp-LPS-O^−^) vaccine. Two months after the last immunization, mice were sacrificed and their spleen cells exposed to OMVs(Bpp-LPS-O^+^), OMVs(Bpp-LPS-O^−^) or to medium alone (negative control). After 72 h of culture, the concentrations of IFN- γ and IL-17 **(A)** were determined in the culture supernatant by ELISA. In both cases, the concentration of cytokine in pg/mL is plotted on the *ordinate* for exposure of spleen cells from non-immunized mice (white bars) or mice immunized with OMVs(Bpp-LPS-O^+^) (black bars) or with OMVs(Bpp-LPS-O^−^) (gray bars) to either medium alone, medium + OMVs(Bpp-LPS-O^+^) or medium + OMVs(Bpp-LPS-O^−^), as indicated on the *abscissa*. The results are expressed as mean values (± standard error) (*n* = 12 mice per group). Significant differences were analyzed for each cytokine between non-immunized and immunized mice (*p ≤ 0.01, ANOVA followed by the Bonferroni *post-hoc* test). **(B)** Effect of passive immunization with spleen cells collected from OMVs(Bpp-LPS-O^+^) or OMVs(Bpp-LPS-O^−^) -immunized mice. Whole spleen cells (2–5 × 10^7^) from OMV-immunized or non-immunized mice were tested as possible vehicles of immune protection by passive transfer to naïve mice (*n* = 12 per group). Twenty-four h after transfer, the mice were infected with *B. parapertussis* AR729 (5 × 10^7^ CFU/40 μL). In the panel, the log_10_ means ± SEM (error bars) of bacteria per lungs is plotted on the *ordinate* for mice receiving spleen cells from non-immunized donor mice or from those immunized with OMVs(Bpp-LPS-O^+^) or with OMVs(Bpp-LPS-O^−^), as indicated on the *abscissa*. The data are the means of 12 mice per group at 7 days postchallenge. The dotted horizontal line marks the lower limit of detection. The results were analyzed by ANOVA followed by the Bonferroni *post-hoc* test.

To investigate whether the immune spleen cells, and more specifically the T-cells, were also involved in the immunogenicity and protection conferred by the OMVs(Bpp-LPS-O^+^) formulation, we performed spleen-cell–activation and –proliferation assays and adoptive-transfer experiments. The results of the activation assays (Figure 4) revealed that 2 months after immunization, OMVs(Bpp-LPS-O^+^) vaccination in contrast to non-immunized mice induced high concentrations of IFN- γ (844 ± 59 pg/mL; Figure 4A) and IL-17 (244 ± 61 pg/mL; Figure 4A). Similar data for both cytokines were detected in mice immunized with OMVs(Bpp-LPS-O^−^) (Figure 4A). These findings strongly indicated that vaccination with any of the two OMV vaccines used here, induced a mixed Th1-Th17 spleen-cell immune-response profile.

To perform adoptive-transfer assays, naïve BALB/c mice were treated either with 5 x 10^7^ intact spleen cells from mice that had been immunized with OMVs(Bpp-LPS-O^+^) or OMVs(Bpp-LPS-O^−^) vaccine 2 weeks before or with spleen cells from non-immunized animals. Twenty-four h later, the mice (*n* = 12 per group) were infected with 5 x 10^7^ CFUs of *B. parapertussis* AR729 and then sacrificed 7 days later to determine the number of CFU counts in the lungs (Figure 4B). The transfer of spleen cells from immunized animals with any of the two OMV vaccines used here resulted in a non-reduction of bacterial colonization, with the CFU counts in the animals that received immune or naïve spleen cells being statistically indistinguishable.

These results indicate that spleen cell-mediated immunity would appear not to play a significant role in the protection against *B. parapertussis* induced by the OMVs(Bpp-LPS-O^+^) vaccine.

### Protection against intranasal *B. pertussis* challenge after vaccination with OMV from *B. pertussis* supplemented with purified *B. parapertussis* LPS-O

**Figure 5:**
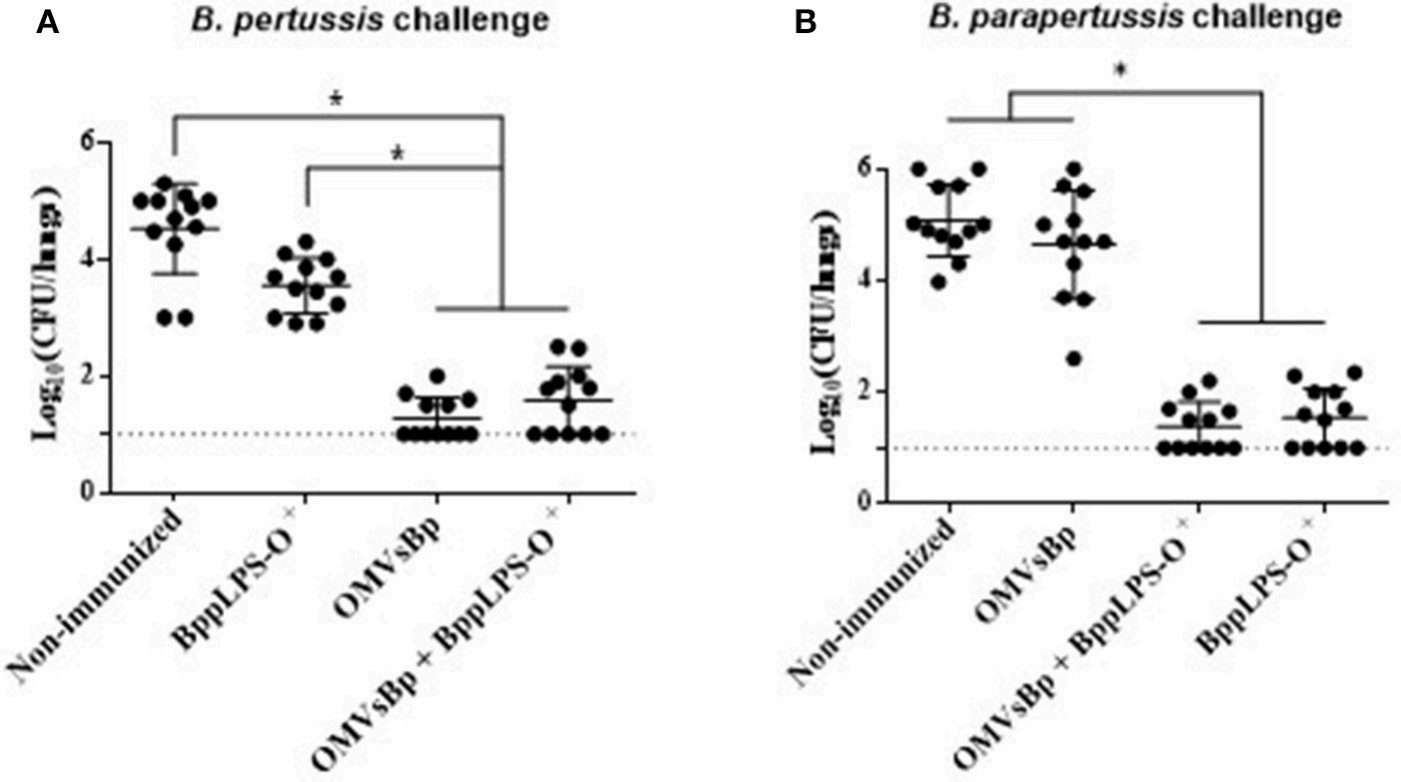
Effect of active intraperitoneal immunization with OMVsBp and/or BppLPS-O^+^ vaccine in the mouse-intranasal-challenge model. Female mice (*n* = 12 per treatment) were vaccinated twice with a OMVsBp + BppLPS-O^+^ vaccine at a 2-week interval. Immunization with OMVs derived from *B. pertussis* or the LPS-O^+^ from *B. parapertussis* alone were used as controls. **(A)** *B. pertussis* Tohama was used as the challenge bacterium (5 × 10^7^ CFUs in 40 μL). **(B)** *B. parapertussis* AR729 was used as challenge bacterium (5 × 10^7^ CFUs in 40 μL). In both panels, the number of bacteria recovered from the mouse lungs, expressed as the log_10_(CFUs) per lungs ± SEM (error bars) is plotted on the *ordinates* for non-immunized mice or for those immunized with a vaccine containing OMVsBp and/or the O-antigen–containing lipopolysaccharide LPS-O+, as indicated on the *abscissa*. Graphs show values for individual mice and the median value (bar, *n* = 12 mice) at 7 days after challenge. The dotted horizontal line marks the lower limit of detection. The asterisks (*) indicate significant differences at a *p* < 0.05 (ANOVA followed by the Bonferroni *post-hoc* test).

In view of the protective capabilities of OMVs derived from *B. pertussis* (OMVsBp) against *B. pertussis* infection and of the purified *B. parapertussis* LPS-O^+^ against *B. parapertussis* challenge, we wanted to evaluate whether a combined experimental vaccine containing OMVsBp plus purified LPS-O^+^ was able to confer a simultaneous protection against both pathogen infections at a comparable level with respect to that of the individual vaccines (OMVsBp vaccine and LPS-O^+^ vaccine). To that end, we analyzed the effect of a double-dose–immunization schedule using a preparation containing OMVsBp plus the purified *B. parapertussis* LPS-O^+^ on the subsequent lung colonization of mice challenged with *B. pertussis Tohama* phase I strain or *B. parapertussis* AR729 (≈10^7^ CFUs in 40 μL; Figure 5A,B). The results were compared with those obtained for mice that had been immunized with each of the two vaccines— OMVsBp and BppLPS-O^+^–exclusively (Figure 5). PBS-injected mice served as a negative control. Significant differences in the lung bacterial counts between the immunized animals and the negative control group were observed (*p* < 0.05; Figure 5). The protection induced by a OMVsBp plus purified LPS-O^+^ vaccine against *B. pertussis* challenge reached the same level as that induced by the OMVsBp immunization alone, with the number of CFUs recovered from the lungs at Day 7 after the challenge likewise dropping by at least 3 orders of magnitude compared to that of the non-immunized mice (Figure 5A). Similar results were observed conversely for *B. parapertussis* challenge: the number of *B. parapertussis* CFUs recovered from the lungs at Day 7 after challenge likewise dropped by at least 3 orders of magnitude in mice immunized either with OMVsBp plus purified LPS-O^+^ vaccine or with LPS-O^+^ vaccine alone compared to the level of infection occurring in the non-immunized mice (Figure 5B).

The results presented in this section thus demonstrated that the vaccines based on OMVs of *B. pertussis* and on the LPS-O^+^ of *B. parapertussis* could be combined without altering the levels of protection inducible by each one independently.

## Discussion

We recently identified a novel vaccine candidate against *B. parapertussis* consisting of the bacterial outer-membrane vesicles (13). In previous work we had presented results on the protection conferred by this OMV-based vaccine formulation against *B. parapertussis* but also against infections caused by *B. pertussis*. In view of the results of Zhang and colleagues on the protective capability of *B. parapertussis* LPS containing the O antigen in its structure (15), we decided to analyze whether OMVs containing this LPS embedded within possessed the same ability to confer protection and, if so, to analyze to what extent that component was responsible for the protective capability (15, 34). In order to achieve these objectives, we carried out comparative studies with a strain of *B. parapertussis* in which we detected the presence of an LPS lacking the O antigen. This detection was potentially both relevant and informative because that strain ATCC 15237 of *B. parapertussis* was isolated by Kendrick and Eldering in 1938 from a human patient with symptoms similar to pertussis (25). To the best of our knowledge, that finding constituted the first description of a human isolate in which the O antigen was not detected. This finding allowed us to perform comparative protection assays, using the intranasal-challenge model in mice, with vaccines based on vesicles derived from the Argentine strain containing the LPS with the O antigen and the ATCC strain lacking that O antigen. These assays enabled us to ascertain the role of the vesicle LPS with O antigen in conferring protection. Thus, while the OMVsBpp containing the LPS with the O antigen adequately protected mice against *B. parapertussis* infection, the presence of LPS without that key antigen in the OMVs was unable to attain the levels of protection afforded by the OMVs(Bpp-LPS-O^+^) -based immunogen. The differential protective capability associated with the presence of the O antigen was confirmed with preparations of purified LPS-O^+^ or LPS-O^−^ as vaccines. Furthermore, the LPS-O^+^ provided a protection against *B. parapertussis* infections even when combined with OMVsBpp containing LPS devoid of the O antigen or with OMVs derived from *B. pertussis* which was previously described as an atractive vaccine candidate againt *B. pertussis* infection containing an important number of protective immunogens (20, 22, 23). Of particular significance in that last formulation combining the OMVs of *B. pertussis* with the LPS-O^+^ from *B. parapertussis* was the ability to protect mice against infections caused by either *Bordetella* species—a feature of considerable relevance to pertussis epidemiology with respect to both *B. pertussis* and *B. parapertussis*.

The results presented here demonstrate that LPS-O^+^ is a protective immunogen even within the internal environment of OMVsBpp.

Antibodies raised against *B. parapertussis* LPS-O^+^ or OMVs(Bpp-LPS-O^+^) efficiently cleared *B. parapertussis* from mouse lungs (Figure 3). In contrast, the immune sera from *B. parapertussis* LPS lacking the O antigen and from OMVs(Bpp-LPS-O^−^) are much less effective in killing the bacteria *in vitro* or in mediating bacterial clearance *in vivo* than sera induced by the formulations containing *B. parapertussis* vesicles with LPS-O+ (Figure 3).

Regarding the bactericidal activity of naïve sera and the sensitivity of *B. parapertussis* to the non-immune complement, the ovine *B. parapertussis* lacking the O antigen was previously reported to be highly sensitive to murine complement-mediated killing *in vitro* (18). Conversely, the O antigen was found to enable *B. parapertussis* to efficiently colonize the lower respiratory tract by protecting against complement-mediated control and clearance (35). The results presented here are in agreement with previous reports (18, 35) demonstrating that *B. parapertussis* strains harboring the O antigen were resistant not only to naïve sera but also to the sera induced by the OMVs(Bpp-LPS-O^−^) (Figure 3F). In contrast, Wolfe et al demonstrated that the complement of immune animals is required for the killing function of serum antibodies against *B. parapertussis* (36). The present study of OMVs(Bpp-LPS-O^+^) -antibody–mediated clearance of *B. parapertussis* furthermore suggests that antibody-opsonized bacteria, in fact, activate the complement cascade, a mechanism of clearance that is in agreement with the one proposed by Wolfe et al. (36).

In these experiments, we also detected that the OMVs derived from *B. parapertussis*, as likewise occurs with those derived from *B. pertussis* (21, 31), were able to induce a Th1-Th17 profile. Nevertheless, in contrast to the OMVs derived from *B. pertussis* (31), we observed that immune spleen cells do not contribute to the protection exhibited by the OMVBpp-based vaccine: rather, that vaccine seems to protect against *B. parapertussis* infection mainly through the induction of a humoral immune response, as was evidenced by the adoptive-transfer experiments. In conclusion, the body of this work has demonstrated that the O antigen constitutes a crucial immunogen—indeed, a *sine qua non*—in the formulation of the OMVBpp-based vaccine.

## Author contributions

DH planned the study, made the laboratory analysis, interpreted data, and drafted manuscript. DB, MG, and MZ planned the study, interpreted data, and revised figures and the manuscript. ME, EB, FC, PM, and CC performed certain experiments and laboratory analyses. All authors approved the final manuscript.

### Conflict of interest statement

The authors declare that the research was conducted in the absence of any commercial or financial relationships that could be construed as a potential conflict of interest.
